# Artificial Intelligence Applications in Medical Devices for Personalized Health Care Solutions: Systematic Review

**DOI:** 10.2196/72410

**Published:** 2026-03-04

**Authors:** Hemanth Ponnambalath Mohanadas, A Manikandan, Ahmad Fauzi Ismail, Nick Tucker, Saravana Kumar Jaganathan

**Affiliations:** 1 Device Development, Abbott Diabetes Care, Alameda, CA, United States; 2 Centre for Material Chemistry, Department of Chemistry, Karpagam Academy of Higher Education, Coimbatore, India; 3 School of Chemical and Energy Engineering, Advanced Membrane Technology Research Centre (AMTEC), University of Technology Malaysia, Skudai, Malaysia; 4 School of Engineering and Physical Sciences, College of Science, University of Lincoln, Lincoln, United Kingdom; 5 Institute of Research and Development, Duy Tan University, 03 Quang Trung, Da Nang, 550000, Vietnam, +84 2363827111; 6 School of Engineering & Technology, Duy Tan University, Da Nang, Vietnam; 7 Biomedical Engineering Research Group, School of Engineering, University of Leicester, Leicester, United Kingdom

**Keywords:** artificial intelligence, personalized medicine, medical devices, digital health, predictive analytics

## Abstract

**Background:**

The integration of artificial intelligence (AI) in medical devices is transforming health care by enabling enhanced personalization and precision medicine. AI-driven medical devices can tailor treatments based on individual patient profiles, including genetic data, medical history, and physiological parameters. This advancement holds the potential to refine therapeutic interventions, improve patient outcomes, and streamline health care delivery. However, challenges such as data quality, algorithmic bias, patient privacy, and regulatory complexities hinder the full realization of AI-driven personalization. By 2030, the global AI in health care market is projected to exceed US $187.95 billion, growing at a compound annual growth rate of 37% from US $15.1 billion in 2022.

**Objective:**

This review aims to explore the scope and impact of AI-driven personalization in medical devices. It seeks to analyze key technological innovations that have enabled AI integration, identify the critical challenges impeding progress, and evaluate strategies to address these challenges. Additionally, it highlights future research directions and innovation opportunities in this evolving field.

**Methods:**

A systematic review was conducted, drawing from scholarly literature, industry analyses, and regulatory advisories. Relevant studies and case examples were analyzed to assess the current applications of AI in medical devices, the barriers to its implementation, and best practices for overcoming these barriers. Ethical, technical, and regulatory considerations were also examined. The review included studies published between 2016 and 2023, covering over 100 peer-reviewed articles and reports.

**Results:**

The review highlights significant advancements in AI-driven medical devices, including applications in diagnostics, treatment personalization, wearable health monitoring, and smart prosthetics. AI-based diagnostic tools have achieved up to 98.88% accuracy in multiclass disease classification from X-ray images and 95% accuracy in insulin injection site recognition. It identifies key challenges such as data security risks, algorithmic biases, regulatory constraints, and integration issues with existing health care infrastructures. Currently, more than 70% of clinical decisions rely on diagnostic tests, yet AI-driven automation could reduce diagnostic delays by up to 50%. Several strategies, including improved data validation techniques, regulatory frameworks for AI approval, and ethical guidelines, were found to be effective in mitigating these challenges. Case studies demonstrate how AI has enhanced medical device functionality and patient outcomes.

**Conclusions:**

AI-driven personalization in medical devices holds immense potential to revolutionize health care, offering more precise, adaptive, and patient-centered solutions. However, successful implementation requires addressing technical, ethical, and regulatory challenges. Emerging technologies such as quantum computing could improve AI-driven medical diagnoses by 10‐20 times in processing efficiency, while blockchain-based patient data management could reduce security breaches by more than 30%. This review serves as a valuable resource for researchers, health care professionals, policymakers, and industry leaders, fostering informed discussions and guiding future advancements in AI-enabled personalized medicine.

## Introduction

### Background

The intersection of artificial intelligence (AI) with medical device technology marks a cornerstone in the evolution of health care, introducing a new epoch where personalization and precision medicine are not just aspirational goals but achievable realities [1]. AI refers to computational systems that perform tasks typically requiring human intelligence, such as learning and decision-making. Personalization in this context is defined as the adaptation of medical devices and interventions to individual patient characteristics, enabled by AI algorithms. AI-driven personalization thus describes the integration of AI into medical devices to dynamically tailor diagnostics, treatments, and monitoring to each patient. This transformative synergy is at the forefront of a health care revolution, offering personalized health care solutions that were once deemed futuristic. Despite these advancements, existing research lacks a comprehensive analysis of how AI-driven personalization is transforming medical devices, particularly in terms of regulatory challenges, clinical adoption, and long-term impact on patient outcomes. While studies have explored AI applications in diagnostics and predictive analytics, a holistic evaluation of AI’s role across the entire medical device spectrum, spanning real-time monitoring, adaptive therapeutics, and regulatory compliance, remains underexplored.

Based on the Precedence Research report, the global AI in the health care market was valued at US $15.1 billion in 2022 and is projected to exceed US $187.95 billion by 2030, growing at a compound annual growth rate of 37% during the forecast period. This substantial growth highlights the increasing adoption of AI technologies in health care, driven by their potential to enhance patient care and operational efficiency. AI-driven personalization in medical devices epitomizes a leap toward a future where medical care is uniquely customized to everyone’s genetic, environmental, and lifestyle specifics, revolutionizing patient care and treatment outcomes [2]. However, despite rapid AI integration, real-world deployment remains constrained by technical limitations, algorithmic biases, and evolving regulatory frameworks. This underscores the need for a systematic evaluation of AI-driven medical devices, addressing both technological breakthroughs and the challenges hindering large-scale implementation.

For instance, additive manufacturing (3D printing) is being harnessed to create bespoke implants and prosthetics tailored to the unique anatomical features of patients, enhancing the effectiveness and comfort of these medical solutions [3]. While promising, the clinical validation of these AI-driven medical solutions remains a major hurdle, with only a fraction of AI-based medical devices receiving regulatory approval due to concerns over accuracy, interpretability, and patient safety.

AI, at its core, involves the development of algorithms and systems that can perform tasks requiring human intelligence, such as learning, reasoning, problem-solving, and decision-making [4]. In the medical device sector, AI technologies are used to process vast amounts of health data to uncover patterns and insights that inform clinical decisions. This includes analyzing genomic data, electronic health records, biometric readings, and real-time physiological data [5]. Advanced AI algorithms and machine learning (ML) techniques are crucial in creating medical devices that can adapt, predict, and respond to the specific needs of individual patients, offering unprecedented levels of accuracy and efficiency [6]. Medical devices integrated with AI range from diagnostic tools capable of early disease detection to therapeutic devices that adjust treatment protocols in real time [7]. These devices not only improve the precision of diagnostics and treatment but also facilitate continuous monitoring and proactive health care management. Applications of AI in medical devices are diverse, including AI-powered imaging tools for cancer detection [8], smart prosthetics that adjust to physiological changes [9], and wearable technologies for real-time health tracking [10]. The regulation of AI in medical devices is a complex but essential aspect to ensure patient safety and efficacy of the devices [11]. Regulatory bodies such as the US Food and Drug Administration (FDA) and the European Medicines Agency are continuously evolving their frameworks to keep pace with rapid advancements in AI technology. These regulations cover various aspects, such as algorithm transparency, data security, patient privacy, and postmarket surveillance [12]. Despite these efforts, regulatory inconsistencies across regions, coupled with the lack of standardized validation protocols for AI-based medical devices, remain a critical research gap. Understanding these challenges is vital for the ethical and effective deployment of AI-driven medical devices.

The scope of AI-driven personalization in medical devices is broad, encompassing a diverse array of applications that promise to enhance disease diagnosis, treatment efficacy, and patient monitoring. For instance, AI-powered imaging tools are revolutionizing diagnostics by providing deeper insights into medical images, enabling early detection of conditions such as cancer with higher precision than ever before [13]. In the realm of treatment, we are witnessing the emergence of smart prosthetics and implants that adapt to patients’ physiological changes and usage patterns, thereby improving functionality and comfort [14]. Furthermore, wearable technologies equipped with AI algorithms are transforming patient monitoring by offering continuous, real-time health tracking and analysis, facilitating early intervention and personalized health management strategies [15].

Despite the great promise of AI in personalizing medical devices, there are many challenges to overcome, including technical, ethical, and regulatory issues. Technical challenges include ensuring the accuracy, reliability, and robustness of AI algorithms, as well as securing high-quality, representative data to train these models effectively [16]. Ethical considerations revolve around issues such as algorithmic bias, which can lead to unequal treatment outcomes, and the imperative to maintain patient privacy and data security in an era of ubiquitous data collection [17]. Furthermore, navigating the regulatory landscape presents its own set of hurdles, as policymakers and regulatory bodies strive to keep pace with the rapid advancements in AI technology while ensuring patient safety and the efficacy of AI-integrated medical devices [18].

This study aims to bridge these gaps by systematically reviewing the role of AI in medical devices, with a focus on its applications, regulatory landscape, and clinical implementation. The objectives of this review are threefold: (1) to explore and categorize AI-driven advancements in medical devices, including their technological underpinnings and real-world use cases; (2) to identify and critically assess key challenges, technical, ethical, and regulatory, affecting AI adoption in medical devices; and (3) to propose strategic recommendations for overcoming these barriers, facilitating a smoother transition of AI-powered devices from research to clinical practice. Our hypothesis is that while AI-driven personalization has the potential to revolutionize medical device functionality, its widespread adoption remains limited due to unresolved challenges in data validation, regulatory approval, and integration into existing health care infrastructures. By systematically analyzing these aspects, this review aims to provide actionable insights for researchers, policymakers, and industry leaders striving to harness AI for personalized medicine.

### Historical Development of AI in Medical Devices

The integration of AI into medical devices has roots that trace back to the mid-20th century, with the advent of computers and early AI research [19]. Initially, these endeavors focused on automating simple tasks and analyzing medical data. A pivotal moment came with the development of expert systems in the 1970s and 1980s, such as MYCIN, a computer-based consultation system designed to assist physicians in diagnosing bacterial infections and recommending antibiotics [20]. These systems laid the groundwork for the incorporation of AI in health care by demonstrating the potential of machine-based decision-making support.

The subsequent decades saw incremental advancements, with AI applications expanding into diagnostic imaging, patient monitoring, and robotic surgery. However, the real explosion of AI in medical devices coincided with the advent of more sophisticated ML algorithms and the exponential growth in computational power and data availability in the 21st century [21]. This era ushered in devices capable of complex analyses and decisions, from predicting patient outcomes to personalizing treatment plans based on individual patient data.

### Basic Principles of AI and ML Relevant to Health Care

AI encompasses a broad range of technologies that enable machines to sense, comprehend, act, and learn. ML, a subset of AI, involves the development of algorithms that can learn from and make predictions or decisions based on data. This is particularly relevant in health care, where ML models are trained on vast datasets of medical records, images, and genetic information to identify patterns and anomalies [6].

Among the diverse techniques under the ML umbrella, 2 approaches are notably significant for personalizing medical devices. Supervised learning, with its basis in training on labeled datasets, proves indispensable in diagnostic realms such as imaging, where algorithms learn from vast databases of prediagnosed images to identify diseases. This training allows machines to recognize complex patterns and anomalies in patient data, making precise predictions or decisions that support clinical diagnoses. On the other hand, unsupervised learning ventures into the analysis of unlabeled data, uncovering hidden patterns without predefined outcomes. This approach is particularly valuable in genomic studies and identifying patient subgroups for targeted treatment approaches, where the absence of labeled data does not hinder the algorithm’s ability to generate insights. Together, these methodologies underscore a transformative shift toward more personalized, data-informed health care solutions, leveraging the intrinsic patterns within vast datasets to cater to individual patient needs [22].

Further advancement within ML, deep learning, delves into complex neural networks with multiple layers, offering profound insights into the intricate datasets typical in health care, such as detailed medical imaging [23]. For instance, deep learning algorithms are used in radiology to enhance the accuracy of detecting abnormalities in X-rays and computed tomography (CT) scans. These algorithms can identify subtle signs of diseases like lung cancer at stages earlier than human radiologists might detect [24]. Another example is the use of deep learning in pathology, where neural networks analyze tissue samples to detect cancerous cells with high precision, significantly reducing the risk of misdiagnosis [25]. In cardiology, deep learning applications include analyzing electrocardiograms to predict the likelihood of heart conditions such as atrial fibrillation or heart failure [26]. These predictive models can alert health care providers to intervene early, potentially preventing serious cardiac events. This progression underscores the pivotal role of AI and ML in not only advancing medical technology but also in shaping the future of personalized health care, illustrating a journey from data to diagnosis that is increasingly nuanced and tailored to individual patient needs.

### The Evolution of Medical Devices From Generic to Personalized Solutions

The journey of medical devices from one-size-fits-all to personalized solutions reflects broader shifts toward precision medicine and patient-centered care [27]. Initially, medical devices were designed for the average patient, with limited ability to adjust to individual needs [28]. However, the recognition that health care efficacy can significantly improve by considering individual differences in genetics, environment, and lifestyle catalyzed the shift toward personalization [29].

Personalized medical devices leverage AI and ML to adapt their functionality to individual patients. For example, wearable devices now monitor health metrics in real-time, adjusting treatment regimens accordingly [30]. Similarly, AI-enabled implants can adjust their behavior based on changes in the patient’s physiological data, optimizing therapeutic outcomes [31].

This evolution is not just technological but also philosophical, representing a shift in health care paradigms toward more holistic, tailored care. It underscores the recognition of the complex interplay between various factors affecting health and the need for treatments that accommodate this complexity [32]. Personalized medical devices, powered by AI, are at the forefront of this shift, offering the promise of health care that is not only more effective but also more attuned to the unique needs and circumstances of each patient.

In summary, the integration of AI into medical devices is a culmination of decades of technological advancement and a reflection of a broader shift toward personalized health care. By leveraging the power of AI and ML, medical devices are evolving to meet the unique needs of individual patients, promising a new era of precision medicine that is more effective, efficient, and patient-centered.

## Methods

### Overview

To comprehensively review the current state of AI-driven personalization in medical devices, a systematic literature search was conducted across multiple databases, including PubMed, IEEE Xplore, Scopus, Web of Science, Embase, and Google Scholar, ensuring broad coverage of relevant studies. To minimize potential gaps, additional sources such as preprint repositories and conference proceedings were also considered. The search strategy incorporated a diverse range of keywords and synonyms, including “artificial intelligence in medical devices,” “personalized healthcare,” “AI-driven diagnostics,” “machine learning in medical technology,” “smart prosthetics,” “ethical considerations in AI-driven healthcare,” and “regulatory frameworks for AI-integrated medical devices.” Boolean operators (AND, OR) were systematically applied, and medical subject heading terms were used in PubMed to enhance precision, with truncation and wildcard operators used where necessary. The search was limited to peer-reviewed studies published between January 2016 and December 2023 to ensure relevance to contemporary developments. While studies were restricted to English-language publications, a cross-verification process was implemented to identify key non-English papers referenced in systematic reviews and meta-analyses, allowing for their inclusion through reliable academic translation tools. This structured search strategy was designed to maximize coverage, ensure methodological rigor, and minimize potential biases, thereby providing a comprehensive and objective review of AI-driven personalization in medical devices.

### Eligibility Criteria

Studies were included if they focused on AI-driven personalization in medical devices, were published in peer-reviewed journals, and provided empirical data, whether quantitative or qualitative. Additionally, studies needed to be available in full text and in the English language. Exclusion criteria encompassed non-English articles, opinion pieces, editorials, letters, studies without accessible full text, and articles unrelated to medical devices or AI. This approach ensured the selection of high-quality and relevant studies for the review.

### Study Selection and Agreement Assessment

The study selection process was conducted by the authors, who were divided into 2 independent groups. Each group systematically screened the titles and abstracts of all identified articles based on predefined inclusion and exclusion criteria. Full-text articles were retrieved for studies deemed potentially relevant. After the initial screening, both groups compared their selections, and any discrepancies were resolved through discussion. In cases where disagreements persisted, a third reviewer (SKJ) was consulted to reach a final decision, ensuring a transparent and unbiased selection process. The overall agreement rate before resolution was 90%, reflecting a high level of consistency in study selection. The detailed study selection process is depicted in the PRISMA (Preferred Reporting Items for Systematic Reviews and Meta-Analyses) flow diagram, which illustrates the number of studies identified, screened, excluded, and included in the review. Following study selection, articles were categorized into three key themes: (1) case studies illustrating state-of-the-art AI-integrated medical devices, (2) applications of AI-driven personalization in various medical devices, and (3) challenges associated with AI-driven medical personalization. This thematic categorization enabled a structured synthesis of findings, ensuring clarity in the presentation of results.

### Data Extraction

Data extraction was performed using a standardized form developed in accordance with the PRISMA-ScR (Preferred Reporting Items for Systematic Reviews and Meta-Analyses for Scoping Reviews) to ensure consistency and methodological rigor. The extracted data included study characteristics (authors, year of publication, and country of origin), AI techniques used (ML models, algorithms, and tools), application in medical devices (type of device and clinical use case), outcomes measured (accuracy, effectiveness, and patient outcomes), and key findings (main results and conclusions). Two authors (HPM and MA) independently extracted data following this predefined protocol to enhance reliability. Any discrepancies were resolved through discussion, and if necessary, by consulting additional authors to achieve consensus. The extracted data were then synthesized according to the predefined thematic categories to align with the structure of the Results and Discussion sections. This structured approach ensured a transparent, reproducible, and comprehensive data collection process.

### Reference Screening

In addition to the database searches, the reference lists of all included articles were manually screened to identify further relevant studies. This snowballing technique ensured comprehensive coverage of the literature.

## Results

### Overview

**Figure 1.**
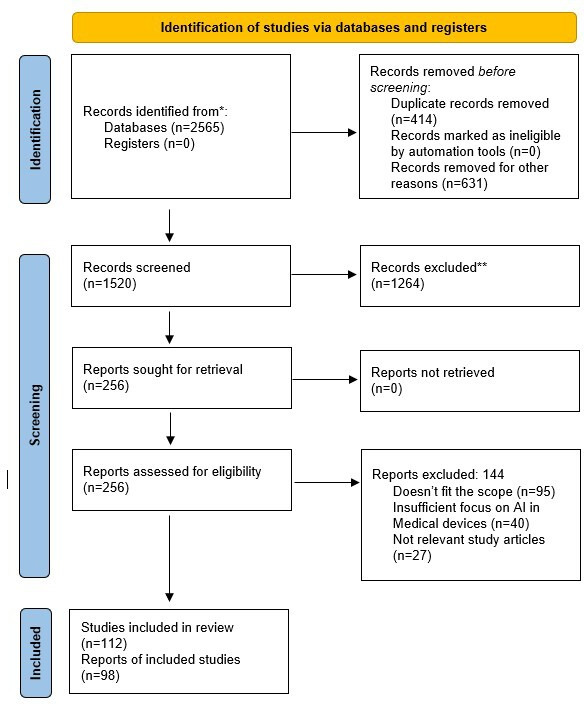
PRISMA (Preferred Reporting Items for Systematic Reviews and Meta-Analyses) flow diagram illustrating the systematic review process for artificial intelligence–driven personalization in medical devices. AI: artificial intelligence.

The results of this review paper are categorized into three major sections: (1) case studies of state-of-the-art AI-integrated medical devices, (2) applications of AI-driven personalization in medical devices, and (3) challenges in AI-driven personalization (Figure 1).

### Case Studies of State-of-the-Art AI-Integrated Medical Devices

#### Smart Insulin Pumps

Smart insulin pumps represent a transformative advancement in diabetes management, addressing many challenges associated with traditional methods that often require frequent manual adjustments [33]. These sophisticated devices use advanced ML algorithms to process real-time data from continuous glucose monitors (CGMs). By analyzing trends and fluctuations in glucose levels, the pumps autonomously adjust insulin delivery, significantly enhancing glycemic control [34]. Adding to this innovation, recent research has successfully applied AI in smart insulin pens, achieving more than 95% accuracy in both detecting proper injection sites and identifying lipodystrophies using a sensor with dual LEDs, thereby promoting better injection practices and reducing tissue complications [35]. Furthermore, another pivotal development includes a smart AI-based self-care device that predicts future blood glucose fluctuations to proactively manage type-1 diabetes, significantly reducing the risk of hypoglycemia and hyperglycemia by intelligently controlling insulin dosage during automatic injections [36]. The first FDA-approved smart insulin pen in 2014, which records and wirelessly transmits insulin dosing data, exemplifies these technological advances, providing clinical data to assist individualized diabetes management and overcoming barriers such as poor adherence and clinical inertia [37]. This dynamic approach not only alleviates the daily burden on patients but also improves their quality of life by minimizing the risk of glucose variability-related complications and enhancing adherence to injection therapy. Studies have shown that such automated systems can lead to better long-term health outcomes, demonstrating a critical evolution in diabetes care technology [38].

#### AI-Powered Prosthetics

AI-powered prosthetic limbs are revolutionizing the field of prosthetics, providing amputees with unprecedented levels of functionality and adaptability. These sophisticated devices integrate sensors and advanced AI algorithms that interpret the user’s muscle signals and movements, allowing the limb to respond in a natural and intuitive manner. As these AI systems interact with the user, they continuously learn and adapt, fine-tuning their responses to better align with individual needs. This technology not only captures and interprets complex muscle signals but also adapts to the user’s unique physiological changes over time, enhancing the effectiveness of rehabilitative practices, as demonstrated in recent studies on surface electromyographic-based systems for hand rehabilitation using AI-powered gesture recognition [39]. Additionally, innovations in lower limb assistive technologies, such as adaptable central pattern generators and personalized motion planning, are enhancing capabilities in gait analysis and stability, crucial for preventing falls and ensuring safe mobility in various environments [40]. Furthermore, AI applications extend beyond mobility aids to the assessment of medical conditions, where tools have significantly improved the evaluation of lower limb radiographs before and after knee arthroplasty, speeding up assessments while maintaining high accuracy [41]. This broad spectrum of AI applications illustrates a transformative impact on patient care, making daily activities and medical diagnostics more efficient and tailored to individual health needs.

#### Diagnostic and Imaging Tools

AI-integrated diagnostic tools have made significant strides, particularly in the field of medical imaging, where deep learning algorithms excel at detecting subtle patterns indicative of diseases like cancer, often outperforming human radiologists in accuracy. These sophisticated tools adapt their analyses based on individual patient data, greatly enhancing diagnostic precision and enabling timely interventions. For instance, deep learning’s integration within imaging modalities has notably advanced pancreatic cancer diagnostics, leading to more precise detection and improved clinical decision-making [42]. Additionally, the application of AI in optical imaging techniques, such as optical coherence tomography and photoacoustic imaging, facilitates high-contrast, low-cost visualization of tumor tissues, thereby enhancing both the detection and theranostics of cancer, which is paving the way for precision oncology [43]. Moreover, the development of a novel deep learning framework for multiclass diagnosis of lung diseases from chest X-ray images shows significant improvements in accuracy and efficiency, exemplifying how AI can transform pulmonary diagnostics and thereby ensure better patient outcomes through enhanced feature extraction capabilities [44]. These advancements underscore the transformative impact of AI in medical diagnostics, revolutionizing early disease detection and treatment strategies, and thereby playing a crucial role in enhancing personalized and effective health care solutions.

#### AI-Powered Humanoids

AI-powered humanoids are revolutionizing various sectors by merging advanced robotics with deep learning technologies to perform complex tasks. These humanoids, equipped with AI, are capable of high-level functions such as speech recognition, comprehensive comprehension, and sophisticated interactions, making them invaluable in industries like health care, education, and space exploration. For instance, AI-based 3D-printed humanoids represent a significant leap forward, offering customizable interactions and decision-making capabilities that mimic human actions [45]. Similarly, AI-powered smart glasses enhance human-robot interaction by allowing dynamic adaptation to varied walking terrains, demonstrating the potential of embedded devices in everyday environments [46]. Furthermore, discussions about responsibly regulating AI to prevent ethical dilemmas and enhance user safety emphasize the need for knowledge about AI’s operational boundaries [47]. In customer service, AI robots are researched for their emotional and functional impact on customer engagement, showcasing their potential to deliver meaningful and personalized service experiences [48]. Moreover, in health care, AI-powered avatars are being evaluated for their acceptance by patients, particularly in mental health services where they can act as assistants to physicians in resource-limited settings [49]. Emotional intelligence in robots, allowing them to recognize and respond to human emotions, is another frontier being explored to make interactions with humanoids more natural and effective [50]. Collectively, these advancements are not just enhancing the capabilities of AI humanoids but are also setting the stage for future innovations that could redefine human-machine collaboration.

#### Other AI-Powered Applications

AI-powered applications are expanding their reach and transforming industries with innovative and practical solutions. In dentistry, the integration of AI and augmented reality is revolutionizing both practice and education, offering new possibilities for enhanced clinical precision and interactive learning environments [51]. Similarly, the application of AI in the fabrication of 3D-printed materials is propelling the creation of patient-specific wearable devices and smart biomedical implants, streamlining manufacturing processes and advancing personalized medicine [52]. In the field of orthopedics, AI algorithms are being used to standardize the measurement of implant alignments in radiography, improving the reliability and efficiency of postoperative assessments [53]. The introduction of AI-powered chatbots in medical consultations offers immediate, personalized medical advice, drastically enhancing patient engagement and health care delivery [54]. Moreover, AI is pivotal in field operations, where it aids in the digitalization and enhancement of inspection rounds, significantly optimizing process monitoring and anomaly detection in industrial settings [55]. Additionally, the development of AI-powered neural implants is opening new frontiers in the treatment of conditions like Alzheimer disease, enhancing communication capabilities and patient care through advanced brain-machine interfaces [56]. Each of these applications mentioned in Table 1 not only highlights the diverse capabilities of AI across various sectors but also underscores the significant impact of intelligent technology in improving operational efficiency, accuracy, and personalized solutions.

**Table 1.** Case studies: artificial intelligence applications in personalized health care devices.

Case studies	Device type	Specific AI^a^ technology	Applications	Outcomes
Exploiting AI to make insulin pens smart: injection site recognition and lipodystrophy detection [35]	Smart insulin pen	Machine learning (one-class SVM^b^)	Diabetes management	Achieved more than 95% accuracy in injection site recognition and lipodystrophy detection, promoting better adherence to injection therapy and improving patient quality of life
Smart AI-based self-care device for patients with diabetes [36]	Insulin pump	AI-based predictive algorithms	Managing type-1 diabetes	Intelligently controls insulin dosage during automatic injection, predicts future blood glucose levels, monitors and reports critical conditions, sends summarized reports to health care centers, maintains a timeline of data and insulin dosages
Empowering hand rehabilitation with AI-powered gesture recognition: a study of an sEMG^c^-based system [39]	Wearable rehabilitation glove	Deep learning (1D-CNN^d^, InceptionTime [an advanced deep learning architecture designed for time series classification])	Hand rehabilitation	Enhanced hand gesture recognition with 90.89% accuracy, aiding in the rehabilitation of stroke patients through mirror therapy and task-oriented therapy. Improved motor function and usability in both clinical and home settings.
The role and efficiency of an AI-powered software in the evaluation of lower limb radiographs before and after total knee arthroplasty [41]	Radiograph analysis tool	AI-powered software (LAMA, U-net-based convolutional neural network)	Evaluation of lower limb radiographs to assess alignment and angles before and after total knee arthroplasty	Good to excellent agreement with expert assessments (ICC^e^=0.78‐1.00), significant time efficiency (twice as fast as experts), but struggled with certain measurements (eg, joint line convergence angle, Mikulicz line, and patients with high BMI)
AI-driven diagnosis of pancreatic cancer [42]	Diagnostic imaging	Deep learning and ML^f^	Early detection and accurate diagnosis of pancreatic cancer	Improved accuracy in diagnosis, early detection, better clinical decision-making, and reduced mortality risk
An advanced deep learning framework for multiclass diagnosis from chest x-ray images [44]	Diagnostic tool	Deep learning (CNN^g^)	Multiclass diagnosis of lung diseases (fibrosis, opacity, tuberculosis, normal, viral pneumonia, and COVID-19 pneumonia)	Achieved superior performance with an accuracy of 98.88%, precision of 0.9870, recall of 0.9904, *F*_1_-score of 0.9887, and AUC of 0.9939
AI enables reliable and standardized measurements of implant alignment in long leg radiographs with total knee arthroplasties [53]	Diagnostic tool	AI algorithm for image analysis	Evaluation of long leg radiographs (LLR) post-total knee arthroplasties (TKA)	The AI algorithm showed excellent reliability (ICC>0.97) and reproducibility (96 % reproducible, 92.1 % reliable) in measuring hip–knee–ankle (HKA), femoral component (FCA), and tibial component (TCA) angles.
Deep neural networks improve radiologists’ performance in breast cancer screening [57]	Diagnostic tool	Deep CNN	Breast cancer screening exam classification	Achieved an AUC^h^ of 0.895; model as accurate as experienced radiologists; hybrid model (AI+ radiologist) more accurate than either alone
Deep learning for the classification of small (≤2 cm) pulmonary nodules on CT^i^ imaging: a preliminary study [58]	Diagnostic tool (CT imaging)	Deep learning (CT-lungNET, a deep learning-based malignancy prediction model)	Classification of small pulmonary nodules on CT imaging	Improved AUROC^j^ (0.85 vs 0.82 for AlexNET), faster processing time, and enhanced diagnostic performance for nonradiologists
AI for cardiac diseases diagnosis and prediction using ECG^k^ images on embedded systems [59]	Diagnostic tool	Deep learning algorithms (MobileNetV2 and VGG16)	Analysis and classification of ECG signals to predict cardiovascular diseases	High validation accuracy of 0.95 for MobileNetV2 and VGG16; after implementation on Raspberry Pi, accuracy slightly decreased to 0.94 and 0.90, respectively. Capable of real-time monitoring using smart mobile tools, such as mobile phones, smartwatches, and connected T-shirts.
Personalized diabetes monitoring platform leveraging IoMT^l^ and AI for noninvasive estimation [60]	Wearable device	Deep neural network (GlucoNet)	Noninvasive real-time blood glucose estimation	The AI-powered platform demonstrated an MAPE^m^ of 17.8 % with 100 % of predictions falling in the clinically acceptable zones A and B of the Clarke-error grid. Clinically validated on over 350 patients, capable of handling large datasets, and does not require frequent calibration.
A predictive machine learning tool for asthma exacerbations: results from a 12-week, open-label study using an electronic multidose dry powder inhaler with integrated sensors [61]	Electronic Multi-Dose Dry Powder Inhaler (eMDPI)	Machine learning (gradient-boosting trees)	Predicting impending asthma exacerbations	The AI model predicted asthma exacerbations with an ROC^n^ AUC of 0.83. The model was based on inhaler use data and inhalation parameters, providing a proactive approach to asthma management by identifying exacerbations up to 5 days in advance.

a AI: artificial intelligence.

b SVM: support vector machine.

c sEMG: surface electromyographic.

d 1D-CNN: One-Dimensional Convolutional Neural Network.

e ICC: intraclass correlation coefficient.

f ML: machine learning.

g CNN: convolutional neural network.

h AUC: area under the curve.

i CT: computed tomography.

j AUROC: area under the receiver operating characteristic.

k ECG: electrocardiogram.

l IoMT: Internet of Medical Things.

m MAPE: mean absolute percentage error.

n ROC: receiver operating characteristic.

### Applications of AI-Driven Personalization in Medical Devices

#### Overview

The integration of AI into medical devices has opened a new frontier in health care, enabling personalized medicine that promises more accurate diagnoses, tailored treatments, and continuous patient monitoring. This section delves into the specific applications of AI-driven personalization across various categories of medical devices, highlighting the transformative impact on patient care and health management.

#### Diagnostic Devices: Improvements in Early Detection and Accuracy

About 70% of clinical decisions rely on diagnostic tests, which play a key role in evidence-based patient care. However, the current method of sending samples to centralized labs often causes delays of several days in getting results. This delay can limit the usefulness of diagnostics and increase patient stress while waiting for important test results [62]. AI-driven diagnostic devices mark a transformative advance in this realm of medical diagnostics, significantly enhancing early detection and improving accuracy. These devices leverage sophisticated algorithms to sift through and analyze complex medical data, including imaging scans [63], blood tests [64], and genetic information [65]. This capability allows them to detect patterns and anomalies that might be overlooked by human evaluators.

In the field of medical imaging, AI algorithms have demonstrated exceptional proficiency. They interpret data from X-rays, magnetic resonance imaging, and CT scans more quickly and accurately than traditional methods. These algorithms excel at detecting subtle abnormalities that are often indiscernible to the human eye, thus facilitating earlier and more accurate diagnoses of conditions such as cancer, cardiovascular diseases, and neurological disorders [66]. The table below (Table 2) summarizes the distribution of FDA-approved diagnostic devices by clinical specialty, underscoring the dominant role of radiology in this field. According to the FDA article released on December 20, 2024, a total of 1016 diagnostic devices have been approved, with an overwhelming 76% (777/1016) categorized under radiology. This statistic highlights the transformative advancements in AI-powered radiology tools, which have played a pivotal role in improving early detection, especially in imaging modalities like X-rays, CT scans, and magnetic resonance imaging. The remaining categories include devices for genetic testing, blood diagnostics, and other fields, reflecting the breadth of AI integration across diverse diagnostic platforms.

**Table 2.** Distribution of US Food and Drug Administration–approved artificial intelligence or machine learning–enabled medical devices across medical specialties [67].

Categories	Number of devices (N=1016), n (%)
Radiology	777 (76)
Cardiovascular	104 (10)
Neurology	42 (4)
Other categories	27 (3)
Anesthesiology	17 (2)
Hematology	17 (2)
Clinical chemistry	9 (1)
Gastroenterology – urology	14 (1)
Ophthalmic	9 (1)

AI-powered diagnostic tools are reshaping medical imaging by surpassing human radiologists in detecting subtle disease markers, particularly in cancer diagnostics. Deep learning models, tailored to patient-specific data, have improved the early detection of diseases like pancreatic cancer and enhanced clinical decision-making [42]. AI also advances optical imaging techniques, such as optical coherence tomography and photoacoustic imaging, by offering affordable, high-contrast tumor visualization, which supports precision oncology [43]. Additionally, AI frameworks for multiclass lung disease diagnosis via chest X-rays have boosted both accuracy and efficiency, transforming pulmonary care [44].

For example, a study comparing the diagnostic performance of 14 radiologists in breast cancer screening with a deep learning model illustrates the benefits of deep convolutional neural networks (CNNs). This study demonstrates that CNNs can significantly enhance radiologists’ performance, achieving an area under the curve of 0.895. Area under the curve is a statistical measure used to gauge the performance of binary classification models, representing the likelihood that the model correctly distinguishes between positive and negative cases. The study also highlights the successful integration of CNNs with radiologists’ evaluations, leading to improved diagnostic outcomes [57]. Similarly, in lung cancer, AI tools applied to low-dose CT scans can identify small pulmonary nodules that may be early indicators of cancer. The precision of AI in these applications not only improves the outcomes for patients through earlier treatment but also reduces the number of false positives and unnecessary biopsies, thereby optimizing health care resources [58]. In ophthalmology, AI-driven tools are used to diagnose diseases like diabetic retinopathy and glaucoma by analyzing retinal images with high accuracy. Various AI systems, including IDx-DR (Digital Diagnostics Inc.), a tool used to diagnose diabetic retinopathy, and EyeArt (Eyenuk Inc.), a system that autonomously analyzes patients’ retinal images, which have been approved for clinical use, have demonstrated high sensitivity and specificity in identifying diabetic retinopathy through color fundus photography [68]. These models are trained on large datasets of retinal images to identify specific patterns, such as microaneurysms [69], hemorrhages [70], and exudates [71], which are indicative of diabetic retinopathy. The AI algorithms can assess the severity of retinopathy by evaluating changes in the retina’s vascular structure, offering a noninvasive method to monitor the progression of diabetes. Similarly, AI applications in pulmonology interpret chest X-rays and CT scans to identify signs of lung cancer at early stages [72]. During the COVID-19 pandemic, the integration of AI-driven diagnostic tools significantly enhanced the detection and management of the virus using imaging and blood tests, as highlighted by several studies. For instance, AI algorithms applied to chest X-rays and CT scans swiftly improved the diagnostic accuracy for COVID-19, enabling better prediction of disease severity and aiding in patient management [73]. In parallel, simultaneously, innovative AI applications analyzing changes in blood parameters demonstrated the potential of routine blood tests in COVID-19 diagnosis, achieving high classification performance and offering a rapid, cost-effective alternative to standard reverse transcription polymerase chain reaction [74,75]. These advancements underline the transformative impact of AI in health care, providing crucial support in diagnosing and managing COVID-19 effectively.

AI algorithms are making significant inroads in the field of genetic testing as well. They analyze genetic data to predict the risk of genetic disorders and conditions swiftly and with high precision. A notable example is the AI-based clinical decision support tool, Fabric GEM (Fabric Genomics, Inc.), which enhances the diagnosis of rare genetic diseases by effectively prioritizing genetic variants and integrating deep phenotyping through clinical natural language processing. This tool substantially improves the diagnostic accuracy and efficiency for rare genetic disorders, as demonstrated in a retrospective cohort study [76]. Similarly, another study reviews AI and ML approaches using gene expression and variant data to advance personalized medicine, highlighting the potential of these technologies to enhance disease diagnosis, risk assessment, and treatment outcomes across diverse populations and a range of diseases, including cancer, cardiovascular, and neurodegenerative diseases [77]. Furthermore, a separate research effort using supervised ML showcases how models such as support vector machines and K-nearest neighbors can be applied to multifactorial genetic inheritance disorders to predict conditions such as cancer, dementia, and diabetes with high accuracy. Support vector machine is a classification technique that finds the hyperplane that maximizes the margin between different classes in the data, while K-nearest neighbors classifies data based on the majority class among the k-nearest samples, providing a straightforward but powerful approach for predictive modeling [78]. Despite these advancements, the deployment of AI in diagnostic devices comes with challenges such as ensuring data diversity to prevent bias, enhancing the interpretability of AI decisions for clinical use, and navigating complex regulatory and ethical considerations [79,80].

#### Therapeutic Devices: Dynamic Treatment Adaptations and Real-Time Monitoring

AI-enabled therapeutic devices are revolutionizing patient care by adapting treatments in real-time, based on continuous monitoring and dynamic feedback. This capability leads to more effective and personalized treatment plans, enhancing overall patient outcomes [30]. By leveraging real-time data analysis, these devices can respond immediately to changes in a patient’s condition, ensuring that treatments are both timely and precisely tailored to individual needs.

One key application of AI in therapeutic devices is in the management of diabetes through AI-driven real-time insulin pumps, which use continuous monitoring and feedback to deliver personalized and effective treatment plans. This approach is underscored by recent research that introduces a closed-loop electronic system using a proportional-integral-derivative controller and WiFi-controlled voltage to automatically adjust insulin levels based on glucose sensor readings, enhancing diabetes management [81]. These types of dynamic management help maintain optimal blood sugar levels, significantly reducing the risk of complications associated with diabetes. Research developed for an AI expert system based on CGM data that not only automates insulin delivery but also facilitates forward-thinking and collaborative health management between sensors and patients [82].

Another significant advancement is in cardiac care, where AI-powered devices such as pacemakers and defibrillators adjust their functioning based on the patient’s activity level and physiological signals [83]. This ensures that the heart operates as efficiently as possible, providing personalized care that adapts to the daily life and needs of the patient, thereby enhancing treatment efficacy and patient safety. Complementing these innovations, a recent study introduces a neural network capable of identifying the manufacturer and model group of cardiac rhythm devices from chest radiographs with exceptional accuracy. This tool, which achieves 99.6% accuracy for manufacturer identification and 96.4% for model groups, outperforms traditional methods used by cardiologists, offering a quicker and more precise approach to managing patients with implanted cardiac devices [84]. Furthermore, another pivotal study uses CNNs to predict the risk of pacemaker-induced cardiomyopathy using clinical data available prior to pacemaker implantation. Achieving an accuracy rate of 75.8%, this model highlights the potential for AI to proactively manage patients’ health, potentially preventing the onset of cardiomyopathy post-implantation [85].

Expanding on this, AI technologies are being increasingly integrated into various cardiac monitoring tools, leveraging advanced algorithms and wireless connectivity to deliver expert-level diagnostics and continuous heart health monitoring. For example, continuous electrocardiogram recordings help redefine arrhythmia phenotypes and predict the success of antiarrhythmic therapies, while wearable activity trackers measure vital physiological indices that can detect irregularities such as atrial fibrillation. Additionally, smartphone apps contribute to reducing response times for sudden cardiac events, showcasing the integration of novel monitoring devices with ML for scalable cardiovascular management. These advancements in cardiovascular monitoring technologies not only promise to improve personalized care but also pose new challenges and opportunities for the future of cardiac health [86].

These AI advancements in cardiac care not only enhance diagnostic and predictive capabilities but also significantly improve the personalized management and safety of patients requiring cardiac rhythm management. Together, these applications of AI in therapeutic devices demonstrate a shift toward more adaptive, responsive medical treatments that promise to improve patient outcomes by integrating sophisticated technology with real-time health data [87]. Supporting this, a study develops predictive models based on mobile clinical data for patients with heart failure, significantly reducing false alerts and improving the management of patient decompensation through telemonitoring systems [88]. These advances support the ongoing development and refinement of therapeutic devices that not only anticipate health needs but also actively engage with them, paving the way for highly personalized and proactive treatment strategies.

#### Wearable Technology: Continuous Health Tracking and Disease Management

AI-enabled wearable technologies are revolutionizing health management by providing continuous tracking and dynamic feedback, allowing for unprecedented personalization in medical care. Equipped with advanced AI algorithms, these devices analyze data from everyday activities to offer personalized health insights and recommendations, enhancing the effectiveness and timeliness of interventions [89]. This capability helps users manage their health more proactively and enables early detection of potential health issues. Recent advancements in wearable health technology have significantly improved the performance of wearable sensors, enabling real-time health monitoring and early disease detection. Additionally, for chronic conditions like hypertension and heart disease, wearable technologies equipped with AI can predict exacerbations, enabling preemptive medical interventions that tailor treatments to the nuances of an individual’s physiological data [90]. This advanced approach not only optimizes patient care but also integrates seamlessly with the broader goals of precision medicine, underscoring the profound impact of AI in enhancing daily health management.

Wearable devices like fitness trackers and smartwatches have become essential tools in everyday health management, capturing a wide array of data such as heart rate [91], sleep patterns [92], and physical activity levels [93]. These devices leverage sophisticated AI algorithms to analyze this wealth of information, providing personalized insights and actionable recommendations. This functionality not only supports users in maintaining or improving their lifestyle but also plays a crucial role in the early detection of potential health issues [10]. Notably, changes in heart rate variability and sleep quality, as measured by these wearables, may indicate stress or cardiovascular issues. A study found that heart rate variability, tracked through wearable devices, effectively predicts the effectiveness of therapeutic interventions for anxiety and depression, underscoring its value in preemptive health measures [94]. This proactive approach to health monitoring highlights the significant role that wearable technology plays in preventive health care, empowering users to manage their well-being with precision and foresight.

**Figure 2.**
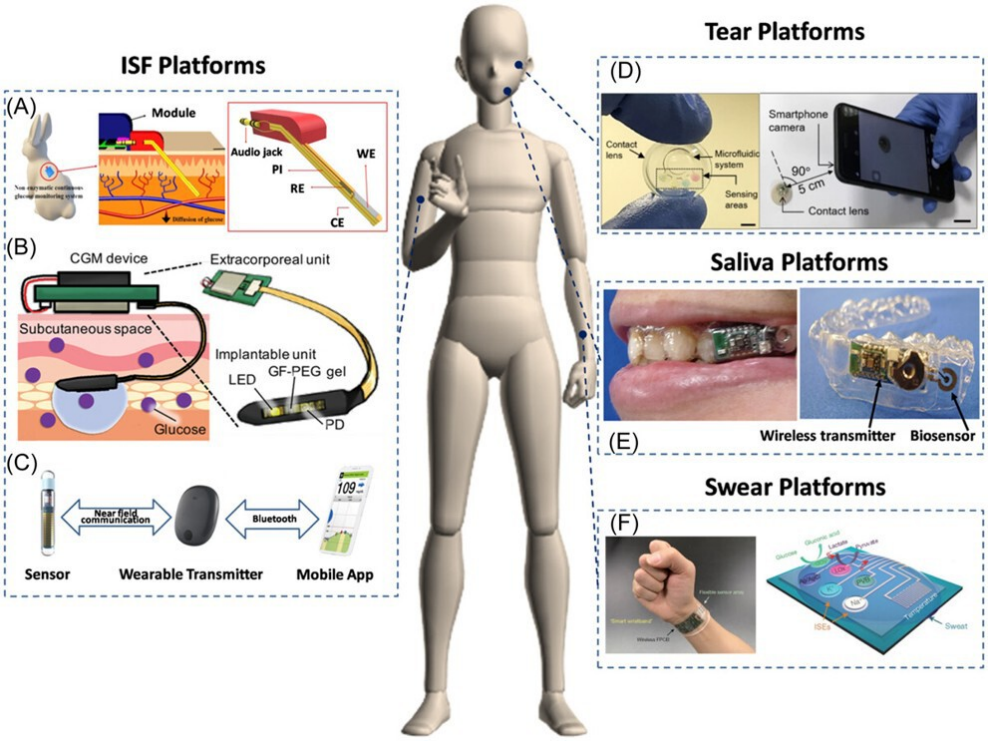
Representative examples of wearable continuous glucose monitoring biosensors [95]. (**A**) An electrochemical nonenzymatic continuous glucose monitoring system. Reproduced from Yoon et al [96] (**B**) A fluorescence-based continuous glucose monitoring biosensor. Reproduced from Sawayama and Takeuchi [97]. (**C**) Commercialized Senseonics continuous glucose monitoring sensor. (**D**) A contact lens biosensor for real-time glucose monitoring. Reproduced from Moreddu et al [98] (**E**) A wearable mouthguard continuous glucose monitoring biosensor. Reproduced from Arakawa et al [99] (**F**) A smart wristband for monitoring glucose in sweat. Reproduced from Gao et al [100]. Full figure reproduced from Jin et al. [95], licensed under CC BY 4.0. CGM: continuous glucose monitoring; GF-PEG: glucose-responsive fluorescent polyethylene glycol; ISF: interstitial fluid.

Figure 2 illustrates various innovative CGM biosensors designed for CGM using different platforms such as interstitial fluid, tears, saliva, and sweat. These wearable devices represent the cutting edge of personalized health monitoring technologies, integrating advanced sensor systems and AI to provide real-time health data, crucial for managing chronic conditions like diabetes [95].

Personalized eHealth programs have demonstrated significant lifestyle improvements among patients with cardiac issues through tailored interventions based on data captured by wearable devices. For instance, the “Do Cardiac Health Advanced New Generation Ecosystem (Do CHANGE 2)” program effectively demonstrates the impact of personalized eHealth interventions, which use wearable technology to promote lifestyle changes and improve the quality of life among patients with cardiac issues [101]. This intervention, documented to significantly enhance lifestyle behaviors through continuous health behavior assessments and personalized feedback, underscores the potential of wearable devices in facilitating substantial health improvements and personalized care. Furthermore, the AI4FoodDB project has innovatively used wearable devices to gather and analyze dietary and lifestyle data, offering groundbreaking personalization in nutritional advice, which significantly impacts lifestyle improvements [102]. Similarly, personalized activity eCoaching, enhanced by AI and semantic ontology, exemplifies how technological advancements can foster more accurate health monitoring and lifestyle recommendations, effectively encouraging healthier behavior patterns [103]. Additionally, real-time models to predict stress-induced blood pressure spikes using wearable devices underline the potential of AI-driven technologies to proactively manage and mitigate health risks [104]. These examples collectively underscore the profound influence of AI-enhanced wearable technology in shaping future health care paradigms, focusing on personalized care and proactive health management strategies.

In the realm of chronic disease management, wearable technologies equipped with AI are particularly transformative. A systematic review of studies involving patients with various chronic conditions demonstrated that wearable devices could facilitate active self-management and real-time health monitoring, thereby playing a crucial role in managing chronic diseases. This data can then be leveraged through AI algorithms to provide actionable insights, which may aid in the early detection and management of potential complications associated with chronic diseases [105]. For instance, wearables have been shown to impact health positively by aiding in the management of diseases like diabetes, where they can monitor and provide feedback on blood sugar levels, or in cardiovascular diseases, where they assist in monitoring heart function [106]. Devices designed for monitoring hypertension and heart disease continuously track vital signs like blood pressure and heart rhythm, transmitting this data in real time to health care providers [107]. AI plays a critical role in this process by analyzing trends and variations in the data to predict potential exacerbations. This proactive approach allows health care providers to offer preemptive medical interventions, potentially reducing hospital visits and improving patient outcomes [108].

This evolution in wearable technology emphasizes a shift from reactive to proactive and personalized medical care. The continuous monitoring facilitated by these devices, combined with AI-driven analysis, ensures that each patient receives care that is tailored not just to a condition but to their unique physiological makeup and lifestyle. This not only enhances patient engagement by providing them with real-time, actionable health insights but also significantly improves the efficacy of interventions, aligning perfectly with the goals of precision medicine [109]. As these technologies advance, they promise to further refine our approach to health management, making preventative care more accessible and effective than ever before.

#### Prosthetics and Implants: Customization and Adaptive Functionalities

The advent of AI-driven prosthetics and implants marks a significant leap forward in the field of medical devices, enhancing the integration of technology and human physiology. These advanced devices harness AI to provide functionalities that closely emulate natural human movements, offering a more intuitive and seamless experience for users [110]. This technological evolution not only improves the quality of life for individuals but also pushes the boundaries of what medical devices can achieve in terms of personalization and performance.

**Figure 3.**
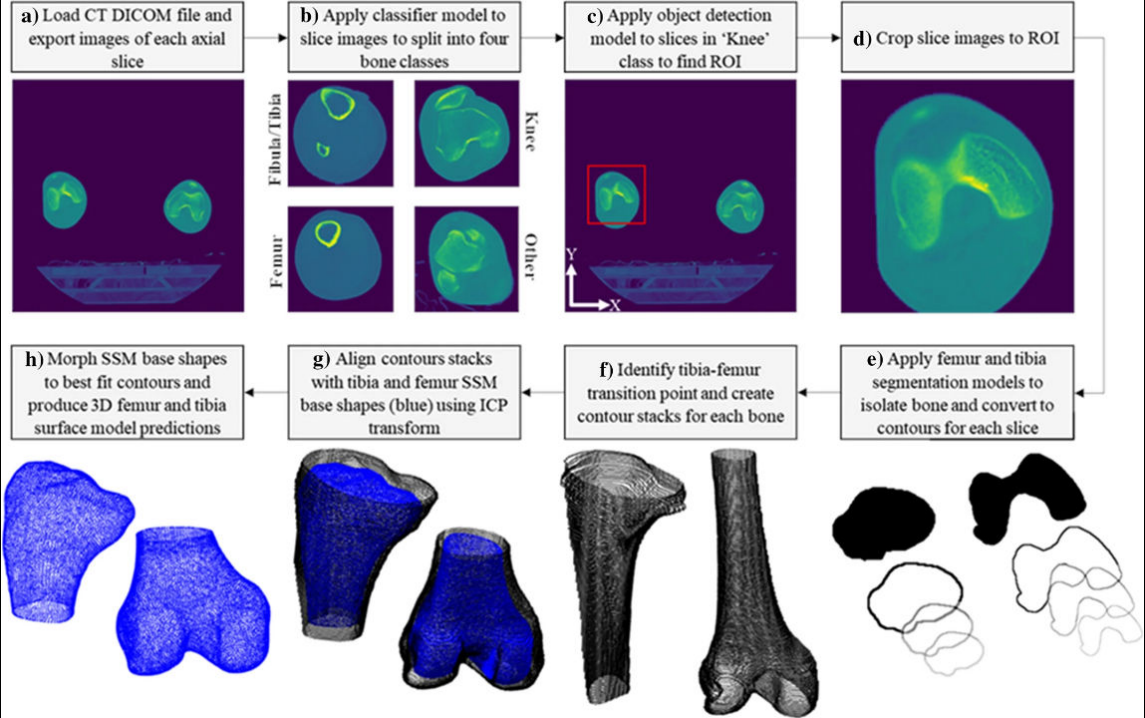
Workflow of computed tomography-3D surface model prediction process for custom total knee replacement design: (**A**) Load digital imaging and communications in medicine, (**B**) classify slices, (**C**) identify region of interest, (**D**) crop slices to region of interest, (**E**) segment femur and tibia bones, (**F**) create contour stacks, (**G**) align contour stacks with statistical shape model base shapes, (**H**) morph statistical shape models to fit contour points and create 3D model predictions [113]. Full figure reproduced from Burge et al. [95,113], licensed under CC BY 4.0. CT: computed tomography; DICOM: digital imaging and communications in medicine; ICP: iterative closest point; ROI: region of interest; SSM: statistical shape model.

In the realm of smart prosthetics, AI plays a critical role in enabling these devices to learn from and adapt to the user’s specific movement patterns. This capability allows for fluid and natural motions, such as a prosthetic arm that adjusts its grip based on the object it is interacting with. This is achieved through advanced sensors and actuators that continually collect and analyze data, allowing the prosthetic to make real-time adjustments. For example, shared control systems in assistive robots predict user intent and react accordingly, enhancing the functionality of the prosthetic while maintaining a balance between user control and automated responses [111]. Similarly, demonstration units for upper-limb prostheses provide valuable insights during the calibration and learning phases, further personalizing the user experience [112]. A recent study developed an automated pipeline using ML methods to design custom total knee replacement implants from CT scans. This pipeline demonstrated high accuracy and repeatability, significantly reducing the time and cost associated with traditional manual processes, highlighting the potential for broader applications in custom medical implants (Figure 3, which illustrates the workflow of the CT-3D surface model prediction process for custom total knee replacement design) [113].

The scope of AI’s impact extends beyond prosthetics to include adaptive implants, which are revolutionizing areas such as orthopedics. Adaptive implants also benefit from AI integration, particularly in orthopedics, where implants can adjust over time to changes in the body or the mechanical loads they must endure. This adaptability can significantly enhance the implant’s longevity and the comfort it offers, making it a vital feature in modern medical practice [114]. For instance, recent advancements in knee arthroplasty involve using ML techniques to automate the customization of stiffness-matched knee implants, ensuring they closely mimic the mechanical properties of the patient’s own bones, thereby improving post-surgical outcomes and promoting natural bone remodeling [115]. Furthermore, new designs in transfemoral prosthetic sockets incorporate a combination of rigid frames and silicone structures that not only offer better biomechanical coupling with the residual limb but also integrate smart technologies for health monitoring and feedback [116]. These AI-enhanced implants can respond to the body’s changes over time, adjusting to shifts in load and movement. In spinal and joint replacement surgeries, AI assisted in the design of implants that align with the patient’s unique biomechanical structure, potentially enhancing the longevity and comfort of these devices [117]. This holistic approach to prosthetics and implants, driven by AI, represents a significant step forward in personalized medical devices, offering enhanced functionality and improved patient comfort through adaptive capabilities.

The potential of AI in prosthetics and implants extends into holistic health management, where these technologies facilitate continuous monitoring and condition management, contributing to preventive health care. Innovations such as electromyography and gyroscope integration in sports training assist systems exemplify how AI-driven devices can enhance physical activities and training effectiveness [118]. Each advancement not only exemplifies the integration of AI in improving device functionality but also highlights the shift toward more personalized, responsive medical care, aligning with the broader goals of enhancing patient outcomes and optimizing therapeutic efficacy.

### Challenges in AI-Driven Personalization

#### Overview

The integration of AI into medical devices heralds significant advancements in personalized health care. However, this integration is not without its challenges, which span technical, ethical, social, and regulatory domains. Addressing these challenges is crucial for the successful implementation and acceptance of AI-driven personalization in medical devices.

#### Technical Challenges

In the realm of AI-driven personalization in health care, technical challenges significantly impact the development and implementation of these innovative technologies. One of the foundational hurdles is the issue of data quality and availability. Effective AI models rely on high-quality, comprehensive datasets, yet health care often struggles with accessing such data [119]. Patient information is frequently dispersed across various institutions and platforms, creating considerable gaps in data necessary for AI training. Additionally, privacy concerns and stringent regulatory frameworks can restrict access to vital data, compounding the difficulty in ensuring data quality, specifically in terms of accuracy, completeness, and relevance, due to varied data collection methodologies and the potential for human error [120,121]. For instance, the integration of wearables in clinical settings necessitates additional validation to confirm their accuracy, reliability, and effectiveness in enhancing outcomes for chronic conditions. Issues such as data privacy, standardization of device functionalities, and integration into existing health care infrastructures also need to be addressed to leverage their potential in chronic disease management [106].

Another significant technical challenge is the development of reliable and valid AI algorithms that perform consistently well across diverse patient populations and clinical environments [122]. Algorithmic reliability is crucial for clinician acceptance and patient safety. Validation of these algorithms demands extensive testing to ensure they deliver accurate predictions and recommendations under various circumstances [123]. However, the opaque nature of some AI models complicates this validation process, leading to concerns about their interpretability and the feasibility of auditing their decision-making processes [124].

Moreover, the successful deployment of AI-driven devices hinges on their seamless integration with existing health care systems and workflows. This integration presents substantial technical challenges, as health care infrastructures are often intricate and composed of outdated software and hardware that may not readily accommodate new AI functionalities [32]. Achieving interoperability between AI systems and electronic health records is essential for real-time data exchange and analysis, yet often faces obstacles due to compatibility issues [125]. These technical challenges must be navigated carefully to realize the full potential of AI in personalizing medical treatment and enhancing patient care.

#### Ethical and Social Challenges

The integration of AI into medical devices, while promising, brings with it a myriad of ethical and social challenges that require diligent attention and strategic management. Privacy and data security concerns are at the forefront of these challenges [126]. The extensive collection, storage, and analysis of sensitive patient data inherent in AI applications necessitate rigorous cybersecurity measures to protect against unauthorized access and breaches [127]. Ensuring the confidentiality, integrity, and availability of this data is not only a technical requirement but also a moral imperative, demanding strict adherence to evolving privacy regulations and standards [120].

Another significant ethical concern is addressing algorithmic bias and fairness. AI systems, inherently dependent on the data with which they are trained, can perpetuate existing biases if the data reflect historical inequities in health care [128]. Such biases could result in AI-driven medical devices performing differently across diverse patient groups, potentially exacerbating health disparities rather than alleviating them [129]. To counteract this, there needs to be a proactive effort in the selection and scrutiny of training datasets and the design of AI models, ensuring they are both inclusive and equitable.

Furthermore, the deployment of AI in health care raises complex issues around patient consent and autonomy. It is essential that patients are thoroughly informed about how their data will be used, the involvement of AI in their treatment, and the implications of decisions made by AI systems [130]. This is particularly challenging given the complexity of AI technologies and the potential for misunderstandings among patients lacking technical expertise. Moreover, care must be taken to ensure that AI-enhanced medical devices support, rather than undermine, patient autonomy, allowing individuals to remain central in making informed decisions about their health care [131].

These ethical and social challenges underscore the need for a balanced approach to the deployment of AI in medical devices, where innovation is matched by an unwavering commitment to ethical standards, patient rights, and social justice. As AI technology continues to evolve, so must the strategies for addressing the ethical implications it brings to the health care landscape.

#### Regulatory and Policy Challenges

Navigating the complex regulatory landscape for AI-enabled medical devices presents a significant challenge as authorities worldwide strive to balance innovation with patient safety. Regulatory bodies are actively developing frameworks that ensure the safety and efficacy of these technologies [12]. This entails rigorous processes for AI development, validation, and ongoing post-market monitoring [132]. The task is particularly daunting due to the rapid technological advances in AI, which require regulators to continually adapt and update guidelines to keep pace with innovation [133].

In addition to regulatory hurdles, the standardization and certification of AI-driven medical devices are critical yet complex issues. Establishing universal protocols for development, testing, and certification is essential to ensure the reliability and interoperability of these devices [134]. However, achieving consensus on these standards involves intricate negotiations among diverse stakeholders from the health care, technology, and regulatory sectors. These standards must not only be robust enough to ensure safety and efficacy but also flexible enough to adapt to future technological innovations [135].

Moreover, the international dimension of AI-driven personalization in medical devices adds another layer of complexity. Global collaboration is necessary to tackle issues like data sharing, privacy protection, and the alignment of regulatory standards [136]. Harmonizing these standards across different jurisdictions can facilitate the widespread adoption of AI technologies in health care [137]. Yet, this requires a concerted effort to reconcile varying regulatory philosophies, health care systems, and cultural attitudes toward privacy and autonomy.

Addressing these regulatory and policy challenges necessitates a multidisciplinary approach that involves experts from health care, technology, ethics, and law. By fostering open collaboration and dialogue among these stakeholders, the sector can effectively navigate the intricacies of AI-driven personalization in medical devices. This collaborative approach is vital for unleashing the transformative potential of AI in health care, enhancing global health outcomes while ensuring compliance with rigorous safety standards.

## Discussion

### Principal Findings

The comprehensive evaluation of AI-integrated medical devices highlights a remarkable evolution in health care technology, spanning multiple domains including diagnostics, therapeutics, prosthetics, and robotics. The results demonstrate how AI-powered solutions are reshaping health care delivery, enabling real-time monitoring, data-driven interventions, and enhanced patient outcomes. In diabetes management, smart insulin pumps exemplify the shift toward precision medicine. These devices leverage real-time data from CGMs and ML algorithms to autonomously modulate insulin delivery, addressing long-standing challenges such as glycemic variability and poor treatment adherence. Complementing this, AI-enabled insulin pens that achieve over 95% accuracy in detecting injection sites and lipodystrophies illustrate how intelligent devices can mitigate complications and support better patient compliance [35]. Further advancements, such as predictive self-care devices, highlight how AI facilitates proactive diabetes management, reducing the risk of hypoglycemia and hyperglycemia [138].

AI-powered prosthetics demonstrate a significant leap in rehabilitation and mobility aids. These systems, particularly those incorporating surface electromyographic-based systems and deep learning frameworks, adaptively interpret complex muscle signals, enhancing patient-robot interaction and optimizing motor function restoration. Innovations in gait analysis and lower limb assistive technologies further contribute to fall prevention and stability in dynamic environments [39]. Beyond mobility, AI-powered tools also enhance clinical workflow, with AI-driven radiograph analysis improving the speed and accuracy of pre- and post-operative orthopedic evaluations.

The diagnostic sector has witnessed transformative developments, particularly with deep learning models outperforming traditional diagnostic approaches. In medical imaging, AI-enabled systems detect cancerous lesions and other pathologies with higher sensitivity and specificity compared to manual interpretation. AI-enhanced frameworks have been pivotal in diagnosing pancreatic and lung cancers, improving early detection rates and clinical decision-making. The successful application of deep learning in chest X-ray analysis, achieving accuracy rates nearing 99%, reflects the capacity of AI to revolutionize pulmonary diagnostics. Moreover, the incorporation of AI into ophthalmology and cardiology diagnostics has led to non-invasive, precise evaluations of diabetic retinopathy and cardiovascular conditions.

### Comparison With Prior Work

In comparison to prior literature, our findings are consistent with earlier studies that highlight the critical role of AI in personalizing medical interventions. For example, the diagnostic accuracy of AI models surpassing traditional radiological assessments aligns with recent meta-analyses showing AI’s superior sensitivity and specificity in medical imaging [43]. Additionally, the effectiveness of AI-powered wearables and biosensors in cardiovascular and metabolic monitoring complements existing literature on personalized chronic disease management [101].

The reviewed case studies build upon previous research by providing concrete evidence of AI’s integration into rehabilitation devices, surgical planning tools, and patient communication platforms, such as AI-powered chatbots. Furthermore, AI-driven technologies are making significant contributions to medical manufacturing, education, and patient communication. AI-enhanced 3D printing supports the creation of personalized implants and wearable devices, while AI-powered chatbots revolutionize patient consultation by delivering timely, accurate medical advice.

Additionally, our review underscores how AI customization in prosthetics and implants contributes to reducing production times and improving biomechanical outcomes, which reflects a growing trend observed in related engineering and biomedical studies. The reviewed studies further elaborate on AI’s role in adaptive implants and intelligent prosthetic sockets equipped with biosensors, offering continuous feedback, improving comfort, and enhancing functional outcomes for users [114].

### Strengths and Limitations

One of the strengths of this review is the comprehensive coverage of AI applications across a broad spectrum of medical devices, ranging from diagnostic imaging to rehabilitation tools and patient engagement platforms. The systematic methodology ensured the inclusion of peer-reviewed studies from diverse health care domains, enhancing the robustness and generalizability of our findings. Additionally, by using a thematic categorization, we provided clarity in synthesizing complex data. The use of a standardized data extraction protocol based on PRISMA-ScR guidelines ensured methodological rigor and minimized bias. Further, the incorporation of snowballing techniques allowed us to capture relevant studies that may have been missed during the initial database searches, contributing to the depth and breadth of our review.

The review has a number of limitations. The restriction to English-language publications may have excluded valuable studies in other languages, potentially introducing language bias. To address this, we conducted cross-verification using key non-English papers referenced in systematic reviews and meta-analyses. Additionally, the study did not fully explore how specific patient demographics, such as age, gender, socioeconomic status, and comorbidities, may influence responses to AI-driven medical devices, which could affect generalizability and clinical relevance. Another limitation lies in the rapidly evolving nature of AI technology, which may render some findings subject to change as new developments emerge. Additionally, while our review emphasizes AI’s potential in health care, real-world implementation challenges such as regulatory compliance, data privacy concerns, and the need for interdisciplinary collaboration remain critical issues for future research.

### Future Directions

Future research should continue to explore the integration of emerging technologies such as quantum computing, blockchain, and AI-driven personalization into health care. These technologies offer opportunities to enhance the precision, security, and scalability of medical devices and health care delivery [139,140].

Quantum computing holds significant promise for advancing the speed and complexity of data processing, which could improve the performance of AI algorithms used in diagnostics, disease monitoring, and predictive analytics [141]. This computational capacity may enable the development of adaptive medical devices that respond dynamically to patients’ evolving health needs [142].

Blockchain technology has the potential to improve data security and integrity by enabling decentralized, tamper-proof health information systems [143]. This could support the development of more trustworthy AI models, particularly in sensitive domains such as patient data management and cross-institutional interoperability [131].

In addition, future research should investigate the role of AI-driven personalized medical devices in addressing global health disparities. Portable AI-enabled diagnostic tools and wearable technologies may help expand health care access in underserved and remote regions [144]. Such innovations could also inform targeted public health strategies, contributing to a more equitable global health landscape. As these technologies become more integrated into health care ecosystems, continued interdisciplinary collaboration will be essential to navigate ethical, regulatory, and technical challenges.

### Conclusions

The integration of AI into medical devices is fundamentally reshaping modern health care by enabling the shift toward personalized and precision medicine. AI-powered innovations, from smart insulin pumps to diagnostic imaging tools and wearable biosensors, are transforming how health care providers diagnose, treat, and monitor patients. These technologies harness advanced ML algorithms and real-time data to tailor medical interventions to individual needs, enhancing treatment efficacy and improving patient outcomes. The dynamic capabilities of AI in automating tasks, recognizing patterns in complex datasets, and adapting to physiological feedback exemplify its critical role in evolving health care delivery toward a more patient-centric model.

The technological landscape continues to expand with advancements in deep learning, natural language processing, and edge computing, which have accelerated the development of AI-enhanced therapeutic and assistive devices. Wearable technologies now enable continuous health monitoring, empowering patients to actively participate in managing chronic conditions such as diabetes and cardiovascular diseases. Meanwhile, AI-driven prosthetics and adaptive implants offer improved functionality and comfort by personalizing device behavior to the user’s biomechanical and physiological characteristics. This fusion of AI with medical devices not only augments clinical workflows but also enhances the quality of life for patients through intuitive, real-time, and highly responsive health care solutions.

Despite these strides, realizing the full potential of AI in personalizing medical devices requires addressing key challenges related to data privacy, algorithmic bias, system interoperability, and regulatory compliance. Ensuring AI’s ethical deployment, maintaining data integrity, and aligning with global safety standards are essential to prevent disparities in health care delivery. Future innovations, including the integration of quantum computing to optimize AI algorithms and blockchain for secure data management, hold promises for overcoming these barriers. As the health care ecosystem embraces interdisciplinary collaboration across technology, ethics, and policy, AI-driven medical devices will increasingly define the future of global health—advancing precision medicine to be more accessible, equitable, and effective for all populations.

## Supplementary material

10.2196/72410 Checklist 1 PRISMA checklist.

## Abbreviations

AI: artificial intelligence
CGM: continuous glucose monitor
CNN: convolutional neural network
CT: computed tomography
FDA: US Food and Drug Administration
ML: machine learning
PRISMA: Preferred Reporting Items for Systematic Reviews and Meta-Analyses
PRISMA-ScR: Preferred Reporting Items for Systematic Reviews and Meta-Analyses for Scoping Reviews
